# The efficacy of the ketogenic diet on motor functions in Parkinson’s disease: A rat model

**Published:** 2016-04-03

**Authors:** Sheida Shaafi, Safa Najmi, Hamed Aliasgharpour, Javad Mahmoudi, Saeed Sadigh-Etemad, Mahdi Farhoudi, Negar Baniasadi

**Affiliations:** 1 Department of Neurology, School of Medicine, Tabriz University of Medical Sciences, Tabriz, Iran; 2 Neuroscience Research Center, Tabriz University of Medical Sciences, Tabriz, Iran; 3 Department of Internal Medicine, School of Medicine, Tabriz University of Medical Sciences, Tabriz, Iran

**Keywords:** Parkinson’s Disease, Pramipexole, Ketogenic Diet

## Abstract

**Background:** The ketogenic diet (KD), high in fat and low in carbohydrate and protein, provides sufficient protein but insufficient carbohydrates for all the metabolic needs of the body. KD has been known as a therapeutic manner intractable epilepsy. In recent years, the effectiveness of KD drew attention to the treatment of some other disorders such as Parkinson’s disease (PD). This study has evaluated the efficacy of KD on motor function in Parkinsonian model of rat and compared it with pramipexole.

**Methods:** A total of 56 male Wistar rats weighing 200-240 g between 12 and 14 weeks of age were randomized in seven 8-rat groups as follows: Control group; sham-operated group; KD group; Parkinsonian control group; KD-Parkinsonian group; pramipexole-Parkinsonian group; and KD-pramipexole-Parkinsonian group. The results of bar test, beam traversal task test, and cylinder task test were compared between the groups.

**Results:** The mean number of ketone bodies had increased significantly in the rats blood after KD. Regarding the results of the triad tests, no statistically significant difference was found between the controls and the sham-operated group. Among the Parkinsonian rats, better results were found in KD groups compared to the non-KD group. The KD enhanced the effect of pramipexole for motor function but did not reach a statistically significant level.

**Conclusion:** The KD reinforced the motor function in Parkinsonian rats in our study. When the diet was combined with pramipexole, the effectiveness of the drug increased in enhancing motor function.

## Introduction

Parkinson’s disease (PD) is the second most common neurodegenerative disease that affects almost 1% of individuals over 60 years of age. First described in 1920,^1^ the ketogenic diet (KD) comprises fat (80-90%), carbohydrate, and protein and metabolically induces a fasting-like condition.^2^^,^^3^ Some studies have shown the usefulness of this diet in PD.^2^^,^^4^^,^^5^

Studies on experimental models of Modeling Parkinson’s disease in primates (MPTP)-induced Parkinsonism have shown that a restriction in glucose intake bolsters resistance of the cells located in the substantia nigra against neurotoxic effects of MPTP and prevents the progression of symptoms associated with PD 2.^5^

Another study, by Vanitallie et al.^6^ on PD patients, shown that the KD for almost 1 month could significantly lessen symptoms and consequently the unified PD rating scale score. Although there is not a consensus on the mechanism underlying the effect of the KD on cerebral pathologies, it seems that the efficacy of ketone bodies in this regard stem from an enhancement of mitochondrial functions and a decrease in oxidative stress^7^^,^^8^ the prevention of the excitotoxicity due to a neurotransmission escalation of excitatory amino acids,^8^ and fending off inflammatory processes and apoptosis.^9^

Pramipexole is a non-ergo dopamine receptor agonist with high affinity toward D2 and D3 dopamine receptors, which has been used for symptomatic treatment of PD in recent years. Noting the mechanism of PD in which there is a decrease in cerebral dopamine levels after degradation of neurons in the substantia nigra, this medication can ameliorate PD motor symptoms through exerting dopamine agonist effects and binding to its receptor.^10^

Because available treatments in PD are usually along with diminished symptoms without affecting progression of the disease and at the same time the potential of causing motor fluctuations, it is essential to use new therapeutic strategies in this regard.^1^

Because there is no available study regarding the effects of the KD on motor symptoms in PD using rat model, this study seeks to examine the effect of this regimen on motor symptoms in rats with 6-hydroxydopamine (6-OHDA)-induced PD and to evaluate the efficacy of this regimen alone or in combination with pramipexole.

## Materials and Methods

In this experimental study, a total of 56 Wistar rats weighing 200-240 g and aged 12-14 weeks were randomized in seven 8-rat groups including controls on a regular diet, sham surgical group, controls on the KD, rats with 6-OHDA-induced PD on a regular diet (negative controls), rats with 6-OHDA-induced PD on the KD for 25 days, rats with 6-OHDA-induced PD on a regular diet and pramipexole for 14 days, and rats with 6-OHDA-induced PD on the KD combined with pramipexole for 14 days. 

This study was performed at Tabriz Neuroscience Laboratory (Iran) from July 2014 to September 2015. The exclusion criteria were an occurrence of any disease during the study period; the expiration of the rats due to intracerebral injections, and no documentation of ketonemia in rats after being on consecutive days of the KD. 

This research was conducted in accordance with the latest ethical regulations for laboratory animal studies issued by Tabriz University of Medical Sciences in terms of providing an appropriate environment for animals, free access to water, and using painless stereotaxic injections.

Rats were first underwent a period of training for a standard bar test, beam traversal task test, and cylinder task test and then randomized in the aforementioned groups. To induce ketonemia, a KD containing medium chain triglycerides (MCTs) oils accounting for a total of 50% of the required calories plus a regular diet for the remaining calories was administered. The MCTs oil was administered orally through standard gavage feeding tubes. 

To ensure induced ketonemia, serum levels of beta-hydroxybutyrate (BHB), as an index for the production of ketone bodies, were measured in both groups including rats on the KD and regular diets. In case of documenting a statistically significant difference between the two groups, an induced ketonemia was confirmed in rats on the KD.^11^^,^^12^

In the present experiment, serum levels of ketone bodies were measured using biochemical kits of BHB at baseline and on the day 14 after being on the KD. 

To induce experimental Parkinsonism, 11 days after the commencement of the KD and regular diets an intranigral injection of 6-OHDA (12 µg in 2 µl normal saline and 0.2% ascorbic acid) was carried out. 30 minutes before, this injection desipramine (25 mg/kg) was injected into the peritoneal cavity to preclude a possible reabsorption of 6-OHDA back into the noradrenergic neurons and subsequent injuries. In the sham surgical group, 6-OHDA was replaced with normal saline.

Finally, 14 days after induction of Parkinsonism the rats underwent a standard bar test, beam traversal task test, and cylinder task test.

***Bar test***

**Figure 1:**
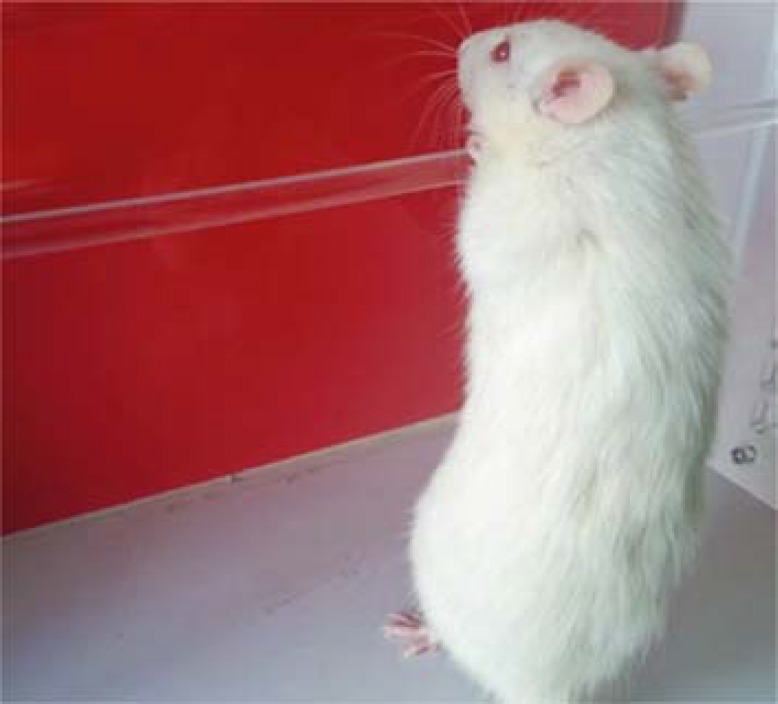
Bar test

As illustrated in figure 1 to perform a standard bar test, the forearms of the animal were placed on a bar (9 mm in diameter) fixed at the height of 9 cm away from the platform of the testing device. The duration of maintaining this position (catalepsy time) was documented. In case of any exploratory head movements or displacement of one or both forearms, the test was considered terminated. 

***Beam traversal task test***

**Figure 2:**
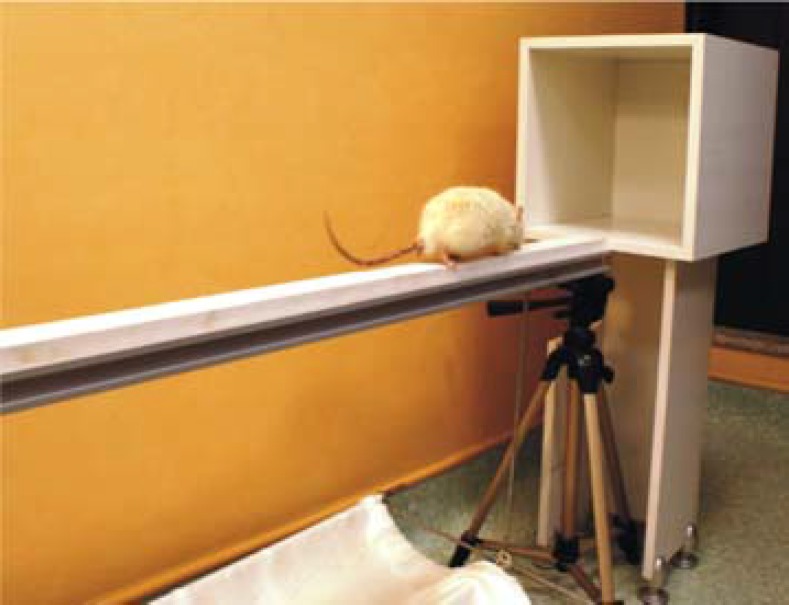
Beam traversal task test

As illustrated in figure 2, 5 cm wide and 1 m long wooden bridge fixed 50 cm above the ground was used. The animal was placed on one end of the bridge and released. The time needed for total crossing the bridge was documented.

***Cylinder task test***

**Figure 3:**
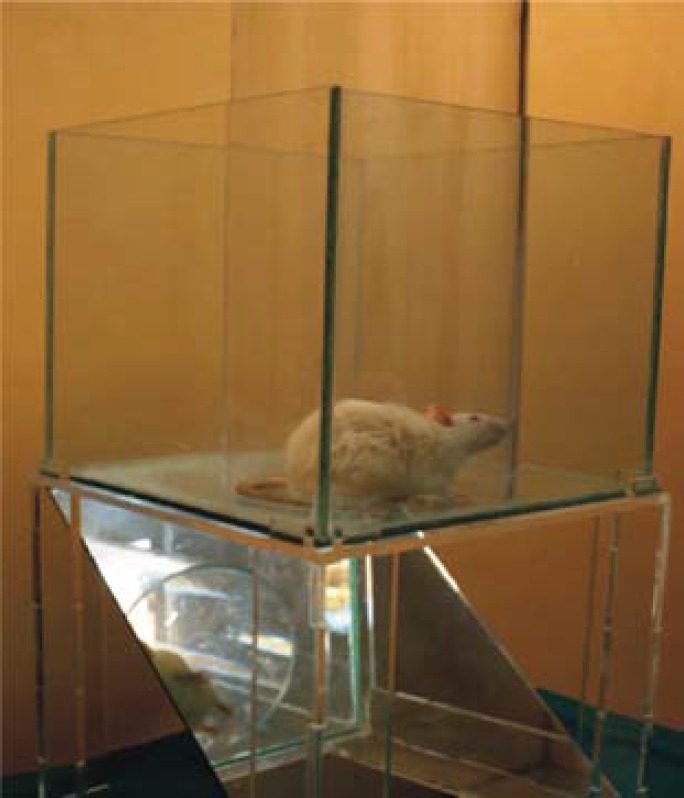
Cylinder task test

As illustrated in figure 3, an animal was placed inside a seven-through glass cylinder, and the total number of forearm contacts with the wall was documented within a 10-minute period. Accordingly, the final score was calculated using the following formula.^13^

Score = 100 × (Number of forearm contacts on the lesion side + 1/2 of total forearm contacts)/total forearm contacts.

The KD was started from the 1^st^ day of experiment and pramipexole (0.3 mg/kg once a day) was administered on the day 12 for 14 consecutive days in relevant groups.

The data were presented as mean ± standard error (SE) of the mean. The SPSS software (version 16, SPSS Inc., Chicago, IL, USA) was used. The one-way ANOVA test coupled with the Tukey post-hoc analysis was employed for comparisons. Levels of serum ketone bodies were compared between groups using the Wilcoxon test. A P < 0.050 was considered statistically significant.

## Results

The mean level of serum ketone bodies in the group on the KD was 0.25 ± 0.03 mmol/L (range: 0.21-0.28) at baseline and 1.83 ± 0.17 mmol/L (range: 1.60-2.10) on the day 14. According to the results of Wilcoxon test, the mean serum level of ketone bodies increased significantly on the day 14 compared to that at baseline (P = 0.010).

***Results of the bar test (catalepsy time)***

**Figure 4:**
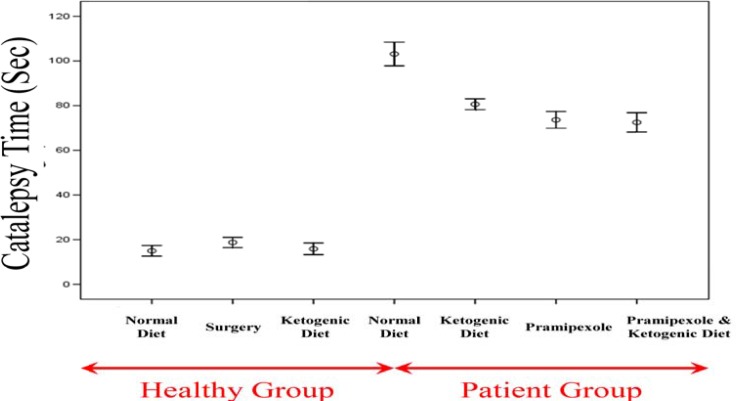
The mean results of the bar test in different study groups

Results of the bar test in different groups were as follows (Figure 4): The control group on a regular diet: 15.00 ± 1.18 seconds (range: 10-20); the surgical sham group: 18.75 ± 1.15 seconds (range: 15-25); the control group on the KD: 15.88 ± 1.30 seconds (range: 10-21); the PD group on a regular diet: 1 03.13 ± 2.65 seconds (range: 95-115); the PD group on the KD: 80.63 ± 1.21 seconds (range: 75-85); the PD group on a diet with pramipexole: 73.63 ± 1.87 seconds (range: 67-80); and the PD group on the KD with pramipexole: 72.50 ± 2.17 seconds (range: 65-80).

Comparing the controls on a regular diet with the sham surgical group showed no significant difference in terms of the mean catalepsy time (P = 0.730). A similar finding was found in the comparison between the controls on a regular diet and the controls on the KD (P = 0.990). Comparing unaffected groups with PD groups revealed that the mean catalepsy time was significantly shorter in the former (P < 0.001 for all paired comparisons). Similarly, the mean catalepsy time was significantly longer in the PD group on the KD compared to that in the remaining three PD groups (P < 0.001 for all paired comparisons). Comparing the PD group on the KD with the PD group on a regular diet with pramipexole did not show a statistically significant difference between the two groups in terms of the mean catalepsy time (P = 0.090). The PD group on the KD, however, had significantly longer mean catalepsy time then the PD group on the KD with pramipexole (P = 0.030). Finally, comparing the PD group on a regular diet with pramipexole with the PD group on the KD with pramipexole did not show a statistically significant difference between the two groups in terms of the mean catalepsy time (P = 0.990).

***Results of the beam traversal task test***

**Figure 5:**
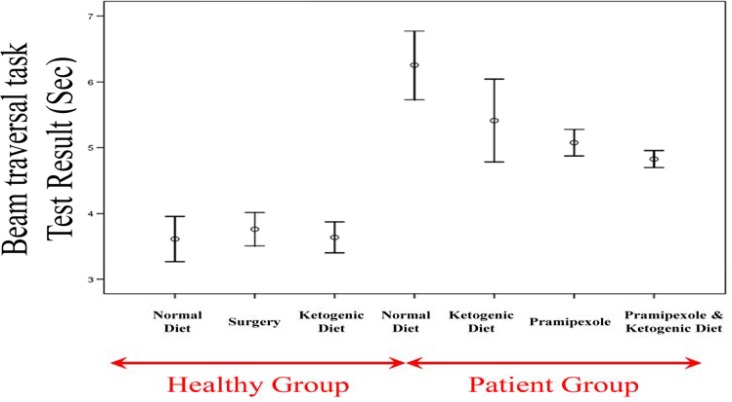
The mean results of the beam traversal task test in different study groups

Results of the beam traversal task test in different groups were as follows (Figure 5): The control group on a regular diet: 3.61 ± 0.17 seconds (range: 3-4); the sham surgical group: 3.76 ± 0.13 seconds (range: 3-4); the control group on the KD: 3.64 ± 0.12 seconds (range: 3-4); the PD group on a regular diet: 6.25 ± 0.26 seconds (range: 5-8); the PD group on the KD: 5.41 ± 0.32 seconds (range: 4-7); the PD group on a diet with pramipexole: 5.08 ± 0.10 seconds (range: 5-6); and the PD group on the KD with pramipexole: 4.83 ± 0.06 seconds (range: 4.5-5).

There was not a significant difference between the controls on a regular diet and the sham surgical group in terms of the mean results of the beam traversal task test (P = 0.990); nor in the comparison between the controls on a regular diet and the controls on the KD (P = 0.990). Comparing unaffected and PD groups, however, showed that the mean results of the beam traversal task test were significantly lower in the former (P < 0.001 for all paired comparisons). Comparing the PD group on a regular diet with the three remaining PD groups showed that the mean results of the beam traversal task test were significantly higher in the former (P = 0.040 in comparison with the PD group on the KD and P < 0.001 for the other two comparisons). Comparing the PD group on the KD and the PD group on a regular diet with pramipexole showed no significant difference between the two groups as to the results of the beam traversal task test (P = 0.860). A similar finding was found in comparisons between the PD group on the KD and the PD group on the KD with pramipexole (P = 0.300), and between the PD group on a regular diet with pramipexole and the PD group on the KD with pramipexole (P = 0.960).

**Figure 6:**
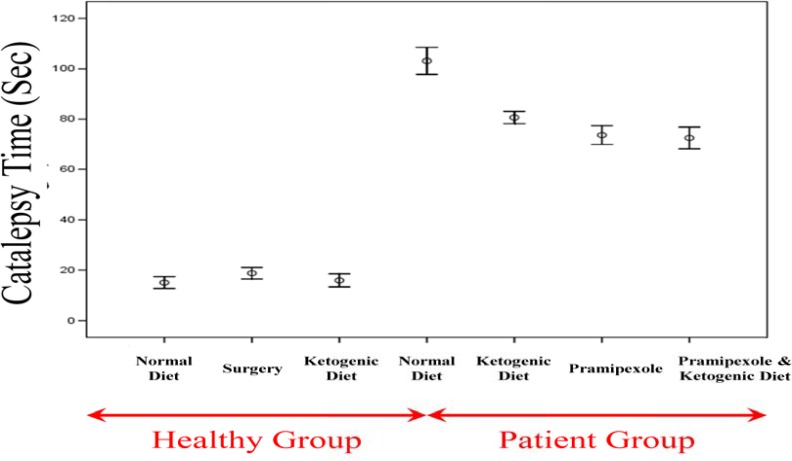
The mean results of the cylinder task test in different study groups

***Results of the cylinder task test***

Results of the cylinder task test in different groups are as follows (Figure 6): the control group on a regular diet: 48.38 ± 1.50% (range: 42-55); the sham surgical group: 50.38 ± 1.48% (range: 45-56); the control group on the KD: 49.88 ± 1.68% (range: 42-56); the PD group on a regular diet: 71.63 ± 1.86% (range: 62-80); the PD group on the KD: 64.13 ± 1.53% (range: 58-70); the PD group on a diet with pramipexole: 60.63 ± 1.10% (range: 57-65); and the PD group on the KD with pramipexole: 58.00 ± 1.48% (range: 52-65). Comparing the controls on a regular diet and the sham surgical group showed no significant difference between the two groups in terms of the mean results of the cylinder task test (P = 0.970). Similarly, no significant difference was found in the comparison between the controls on a regular diet and the controls on the KD (P = 0.990). Comparing the unaffected and PD groups showed significantly lower mean results of the cylinder task test in the former (P < 0.001 for all paired comparisons). In addition, comparing the PD group on a regular diet with the remaining three PD groups showed significantly lower mean results of the cylinder task test in the former (P = 0.020 in comparison with the PD group on the KD and P < 0.001 for the other two comparisons). There was no significant difference between the PD group on the KD and the PD group on a regular diet and pramipexole in terms of the results of the cylinder task test (P = 0.670). There was also no significant difference between the PD group on the KD and the PD group on the KD and pramipexole in this regard (P = 0.090). Finally, there was no significant difference between the PD group on a regular diet with pramipexole and the PD group on the KD with pramipexole regarding the results of the cylinder task test (P = 0.890).

## Discussion

Nutritional and metabolic therapeutic strategies have been tried in a wide range of neurologic diseases such as epilepsy, headache, neurotrauma, Alzheimer’s disease, sleep disorders, brain cancer, autism, pain, multiple sclerosis (MS), and PD. The related incentive possibly originates from the ineffectiveness of available pharmacologic treatments in many of such diseases, as well as a general inclination toward more natural products.^14^

The present experimental study on rats showed that the KD is significantly effective in promoting motor functions in PD animals and at the same time, it may enhance the therapeutic effects of concomitantly administered pramipexole, albeit in an insignificant fashion.

In accordance with these findings, using a standard scale, Vanitallie et al.^6^ showed that the KD was able to exert beneficial effects in 5 out of 7 PD patients.

One of the major limitations in the current study was using a small sample size that was unable to exclude the placebo effect. In a study by Tieu et al.,^5^ the injection of BHB acid also effectively prevented MTPT-induced neurodegenerative and aging processes in dopaminergic cells located in rat brain.

In another animal model used by Cheng et al.^15^ the employment of ketone bodies protected dopaminergic neurons located in the substantia nigra against 6-OHDA-induced neurotoxicity. In a study by Yang and Cheng,^16^ the anti-inflammatory effects of ketone bodies were confirmed using a model of MPTP-induced neurotoxicity.

In a similar experiment by Beckett et al.^17^ using a rat model of Alzheimer’s disease, in conformity with our findings the KD managed to significantly improve some aspects of motor functions. In this study, all aspects of motor functions of the examined animals were not influenced equally. Although the underlying cause of this finding is not clear, it seems that various muscular groups are affected unequally by the KD (for example the quadriceps muscles vs. the paw flexors).

In another series by Brownlow et al.,^18^ there was also a considerable improvement in motor functions in Alzheimeric rats using the KD. Ruskin et al.^19^ also showed that the administration of the KD could increase motor capabilities of animals with induced Huntington’s disease (HD).

Although the exact neuroprotective property of the KD is not known, some hypotheses have been suggested. The two major aspects in treating with the KD is an increase in the production rate of ketone bodies in the liver and a decrease in serum glucose levels. An increased level of ketone bodies is assumed to be related to fatty acid oxidation. Certain polyunsaturated fatty acids such as arachidonic acid (AA), docosahexaenoic acid (DHA), and eicosapentaenoic acid (EPA) may regulate the stimulatory properties of the neural sheaths through inhibiting voltage-dependent calcium and sodium channels, decreasing inflammation and inducing mitochondrial uncoupling proteins that could lead to production of reactive oxygen species (ROS).^14^ Ketone bodies themselves may also bear neuroprotective properties.^2^

This effect develops through increasing the levels of adenosine triphosphate and decreasing ROS production via enhancing nicotinamide adenine dinucleotide hydrogen (NADH) oxidation and preventing mitochondrial permeability change. Along with other enhanced bioenergetics pathways, ketone bodies are able to stimulate mitochondrial biogenesis and stabilize synaptic functions. The second major biochemical feature of ketone bodies is diminishing glycolytic flow. This condition is the main feature in calorie intake limitation that could elongate the life of various species including primates. It seems that the observed neuroprotective effect is due to a diminished incidence of the brain-derived neurotrophic factor and its principle receptor the tyrosine kinase B, an improved mitochondrial function, diminished oxidative stress, a compromised activity of pro-apoptotic factors, and the prevention of inflammatory mediators such as interleukins and tumor necrosis factor-alpha.^14^

Possible mechanisms involved in the effectiveness of the KD in relieving PD symptoms could be summarized as follows:

- Providing an efficacious source of energy, which is capable of preventing local hypometabolism in the brain
- Diminished oxidative injury
- Increasing mitochondrial biogenesis pathways
- Exploiting ketone capacities to bypass a failure in the Complex I activity.^20^

Noh et al.^21^ showed that one of principle mechanisms of the KD in preventing neurodegeneration was via impeding neural apoptosis by caspase-3.

The early pathophysiology of PD is an excitotoxic degeneration of the dopaminergic neurons located in the substantia nigra. On the basis of some findings, ketone bodies may bypass defects in mitochondrial Complex I activities, which are possibly involved in the pathogenesis of PD.^6^ Therefore, it seems that mitochondrial abnormalities play a pivotal role in this regard.^22^

Kashiwaya et al. used an analog of heroin to destroy dopaminergic cells. The suggested mechanism involved in this process was the blockade of a mitochondrial NADH dehydrogenase multi-enzyme complex.^4^ Accordingly, much of previous studies on the role of the KD in PD have been focused on this aspect of the disease (i.e., mitochondrial abnormalities).^23^^-^^27^ For example, in a study by Kashiwaya et al.^4^ on an animal model of PD induced by MPTP, the administration of BHB reduced mitochondrial respiration cycle lesions, which are usually induced by the used toxin.

In a study by Kim et al. the protective effects of ketone bodies against mitochondrial respiratory complex lesions developed by inhibitors of complex I (rotenone) and II (exogenous 3-nitropropionic acid) were examined. They suggested their findings as potential neuroprotective mechanisms of ketone bodies in PD.^7^

Nevertheless, the current study is one of the rare experiments that examine the effect of the KD on one aspect of PD in a rat model. The observed results are promising and could pave the way for further human studies. For instance, recently some commercial treatments have been available which are based on development and exacerbation of ketonemia such as a formulation using medium-chain triglycerides. It is not clear whether such treatments are effective against PD as much as they are against Alzheimer’s disease. Further studies are needed in this regard.^28^

## Conclusion

According to the findings of the present study, since the KD is effective in improving motor function in PD rats further human studied are recommended in this regard.

## Notes:

**How to cite this article:** Shaafi Sh, Najmi S, Aliasgharpour H, Mahmoudi J, Sadigh-Etemad S, Farhoudi M, et al. The efficacy of the ketogenic diet on motor functions in Parkinson’s disease: A rat model. Iran J Neurol 2016; 15(2): 63-9**.**
